# Timing of supplementation of selenium and isoflavones determines prostate cancer risk factor reduction in rats

**DOI:** 10.1186/1743-7075-5-31

**Published:** 2008-11-10

**Authors:** Jessica R Tolman, Edwin D Lephart, Kenneth DR Setchell, Dennis L Eggett, Merrill J Christensen

**Affiliations:** 1 Department of Nutrition, Dietetics and Food Science, Brigham Young University, Provo, Utah 84602, USA; 2 Department of Physiology, Developmental Biology and the Neuroscience Center, Brigham Young University, Provo, Utah 84602, USA; 3 Department of Pediatrics, Children's Hospital Medical Center, Cincinnati, Ohio 45229, USA; 4 Department of Statistics, Brigham Young University, Provo, Utah 84602, USA; 5 Cancer Research Center, Brigham Young University, Provo, Utah 84602, USA

## Abstract

**Background:**

High dietary intake of selenium or isoflavones reduces risk factors for prostate cancer. We tested whether combined supplementation of these two dietary components would reduce prostate cancer risk factors in rats more than supplementation of each component individually.

**Methods:**

Male Noble rat pups were exposed from conception to diets containing an adequate (0.33–0.45 mg/kg diet) or high (3.33–3.45 mg/kg) concentration of selenium as Se-methylselenocysteine and a low (10 mg/kg) or high (600 mg/kg) level of isoflavones in a 2 × 2 factorial design. Pups consumed their respective diets until sacrifice at 35, 100, or 200 days. Male Noble rat breeders, whose exposure to the diets began after puberty, were sacrificed at 336 days. Rats were weighed biweekly. Blood was collected at the time of sacrifice and body fat and prostates were dissected and weighed. Serum levels of leptin, IGF-1, and testosterone were determined using ELISA kits. Serum levels of isoflavones were assayed by GC/MS. Liver activity of selenium-dependent glutathione peroxidase 1 was measured as an indicator of selenium status.

**Results:**

Serum isoflavone concentrations were nearly 100-fold higher at 35 days of age (1187.1 vs. 14.4 ng/mL, mean ± SD) in pups fed the high vs. low isoflavone diets, and remained so at 100 and 200 days, and in breeders. There were no dietary differences in liver glutathione peroxidase activity in pups or breeders. High isoflavone intake significantly (p = 0.001–0.047) reduced body weight in rat pups from 35 days onward, but not in breeders. Body fat and leptin were likewise significantly reduced by high isoflavones in pups while effects in breeders were less pronounced but still significant. High intake of Se and isoflavones each decreased serum IGF-1 in pups at 100 and 200 days, but not in breeders. No consistent dietary effects were observed on serum testosterone or relative weights of prostates. In pups, the combination of high isoflavones and high selenium produced the lowest weight gain, the lowest serum leptin, and the lowest serum IGF-1 concentrations of all four diets.

**Conclusion:**

Combined intake of high selenium and high isoflavones may achieve greater chemopreventive effects than either compound individually. The timing of supplementation may determine the significance of its effects.

## Background

Prostate cancer is the most frequently diagnosed cancer and the second leading cause of cancer deaths in American men. The American Cancer Society projects that there will be 186,320 new cases of prostate cancer in the U.S. in 2008, and 28,660 deaths from this disease [1]. The high occurrence and long latency period of prostate cancer make it a good candidate for intervention and chemoprevention by diet and other means.

Many host factors, including the hormonal profile to which tumors are exposed, contribute to their growth and spread. Obesity and excess body fat increase risk of several cancers, including that of aggressive prostate cancer [2]. High plasma concentrations of insulin and insulin-like growth factors (e.g. IGF-1) are likewise associated with increased risk [3,4]. Serum leptin concentrations show a direct association with prostate cancer risk [5-7], and testosterone is a well-known promoter of the growth of both normal prostate tissue and prostate tumors [8,9]. Several of these factors are responsive to dietary manipulation.

Selenium (Se) and soy products containing isoflavones are among the most promising dietary constituents whose intake may reduce risk for prostate cancer [10,11] and among the most popular supplements taken by men in a recent prostate cancer trial [12]. In their review of diet in the development and progression of prostate cancer, Chan et al. [13] found the evidence more convincing for a protective effect of the essential trace element Se than for any other dietary component. Several lines of evidence confirm the chemopreventive efficacy of high dietary Se intake or status against prostate cancer [14-18]. The World Cancer Research Fund and American Institute for Cancer Research [19], in their recently released summary, concluded that "The evidence from cohort and case-control studies is consistent, with a dose-response relationship. There is evidence for plausible mechanisms. Foods containing selenium probably protect against prostate cancer."

Molecular targets for Se have been identified [15,20,21] and mechanisms for its protective effects have been proposed [22-27]. These mechanisms include alteration of various aspects of steroid hormone metabolism [27,28].

Prostate cancers rates are particularly low in Asian countries where the typical diet contains high levels of soy products [29]. In contrast, the highest rates of prostate cancer are seen in countries where the so-called "Western diet" is the norm and intake of soy products is very low [19,30-33]. Many investigators have attributed the inverse correlation between soy intake and prostate cancer to the estrogenic isoflavones found in soy [34-36]. High intake of soy products has been correlated in epidemiologic [37,38] and case control studies [39] with reduced risk for prostate cancer, although not all studies are in agreement [40,41]. Mechanisms for the chemopreventive effects of soy have been reviewed [42,43]. Effects of phytoestrogen consumption may depend in part on genotype [44]. Much of the mechanistic work related to soy consumption has focused on individual isoflavones supplemented in cell culture medium or provided to animals by injection or diet. Among other effects, dietary isoflavones have been shown to modify body weight [45,46], deposition of body fat [46,47], leptin [46,47] and insulin [47,48] levels, and various aspects of steroid hormone metabolism [47,49,50].

In this study we investigated the effects of combined dietary supplementation of Se and isoflavones to determine if risk factors for prostate cancer might be reduced more by their combined use than by supplementation of either dietary component individually. The molecular mechanisms by which Se and soy isoflavones act individually are being critically examined by many investigators, but the potential chemopreventive efficacy of combining supplementation of these two dietary components remains to be explored.

## Methods

All procedures involving animals were approved by the BYU Institutional Animal Care and Use Committee. Male and female Noble rats, aged 35–42 days were purchased from Charles River Laboratories (Wilmington, MA). Females were housed individually in shoebox cages and males were housed individually in hanging wire cages. For breeding, females were placed with males in the hanging wire cages, which facilitated identification of the vaginal plug after mating. Following the appearance of the plug, females were returned to their shoebox cages. All cages were kept in a temperature- and light-controlled room (12 h, 0600–1800, light:12 h, 1800–0600, dark). Animals were given free access to food (as described below) and tap water.

Four males and eight females were assigned to each of the four experimental diets. After consuming their respective diets for at least 30 days males and females within each dietary group were bred to produce pups whose exposure to the diets began at conception. Dams continued consuming their respective diets through pregnancy and lactation. Male pups were weaned at 21 days of age to the same diets consumed by their dams, and used as experimental subjects. Male breeders continued to consume their respective diets until they were sacrificed at approximately 336 days of age. Thus, continued feeding of breeders made possible comparisons between animals whose exposure to the diets began at conception and those whose exposure began after sexual maturity.

Rats were fed one of two basal stock diets, with or without a supplement of Se, in a 2 × 2 factorial design. Stock diets were 1) a phytoestrogen-reduced formulation (Zeigler Bros., Inc., Gardners, PA; Phytoestrogen Reduced Diet I); or 2) a standard soy-based laboratory chow (8604, Harlan Teklad, Madison, WI). Previous analysis showed these formulations to contain approximately 10 (Low) and 600 mg (High) isoflavones/kg diet, respectively [51]. Product data sheets reported that the basal diets contained 0.33–0.45 mg Se/kg diet (Adequate Se). Half of the diets were supplemented with 3.0 mg/kg Se as Se-methylselenocysteine (a generous gift from Kelatron Corp., Ogden, UT; High Se). A detailed analysis of the two basal stock diets is shown in Additional file 1.

Rats were weighed biweekly. All rats were weighed immediately prior to sacrifice. Eight pups in each dietary group were killed at 35, 100, or 200 days of age by decapitation. Breeders were sacrificed at approximately 336 days of age. Blood was collected, allowed to clot, and centrifuged at 1000 × g for 10 minutes to separate serum from clotting factors and cells. White adipose tissue was dissected from the abdomino-pelvic cavity of 200 day old rats and breeders, and weighed. For pups and breeders, testis and ventral prostate lobes were dissected and weighed. Livers were also dissected and flash frozen in liquid nitrogen for subsequent analysis of cellular Se-dependent glutathione peroxidase 1 (EC 1.11.1.9; GPX1) activity.

Analysis of serum isoflavone content was performed as described previously [52]. Briefly, phytoestrogen concentrations were analyzed for each dietary treatment group via gas chromatography/mass spectrometry (GC/MS). This was performed by liquid-solid extraction and liquid gel chromatographic techniques to isolate the phytoestrogen fractions using standards (internal controls) to validate the assay. As a measure of Se status, activity of GPX1 was assayed in cytosolic fractions from livers of all animals at each time point in each of the four dietary groups, and in breeders, as previously described [53].

Serum hormone levels were assayed using commercially available ELISA kits according to the manufacturers' directions. IGF-1 was measured using R&D Systems Mouse IGF-1 Immunoassay Quantikine kit (#MG100, Minneapolis, MN). Intra-assay precision of this kit ranges from 3.3–5.6% (CV) and inter-assay precision ranges from 4.3–9.0% (CV). Testosterone was assayed using an ELISA kit from Fitzgerald Industries International Inc. (#RDI-RE52151, Concord, MA). Intra-assay variation for this kit ranges from 3.28–4.16% (CV) while inter-assay variation is between 4.73–9.94% (CV). The mouse/rat leptin ELISA kit used was from BioVendor (#RD291001200, Candler, NC). For this kit, reported intra-assay variability (CV) is 2.2%, while inter-assay variability (CV) is 3.4%. Microplates were read on a FLUOstar Optima microplate reader (BMG Labtech, Durham, NC).

Statistical analysis was performed by analysis of variance (ANOVA) using the General Linear Model in SAS (SAS Institute Inc., Cary, NC) to determine main effects of Se and isoflavones, and their interactions. This was followed by Fisher's and Tukey's pairwise comparisons to determine significance of differences between dietary groups. Differences for which p values were < 0.05 were judged statistically significant.

## Results

### Nutritional status indicators

Serum isoflavone levels (genistein + daidzein + equol) were significantly higher in rats fed the high isoflavone diets, even at the earliest time point measured. At 35 days serum concentrations averaged 1235 ± 117 (mean ± SD) and 1140 ± 275 ng/mL in rat pups consuming high isoflavone diets, with and without a 3.0 mg/kg Se supplement, respectively. In contrast, serum concentrations in pups fed the low isoflavone diets, with and without the Se supplement, averaged 11.9 ± 4.1 and 16.8 ± 2.5 ng/mL, respectively. Supplemental Se had no effect on serum isoflavone levels. These differences persisted at 100 days and at 200 days in pups, and comparable differences were seen in breeders (1419 ± 135 vs. 17.3 ± 3.2 ng/mL, in rats fed 600 vs. 10 mg isoflavones/kg diet, respectively). The serum isoflavone concentrations in rats fed 600 mg/kg were similar to those seen in adults in Asian countries while the serum levels in rats fed only 10 mg/kg were comparable to those in persons consuming diets typical of Western countries [31,54].

There were no statistically significant differences due to diet at any time point, in pups or in breeders, in the activity of hepatic GPX1. This was expected since all diets provided a Se concentration higher than needed to maximize GPX1 activity in rat liver [55]. Mean activities at different time points in the four dietary groups ranged from 461.6–868.7 mU/mg protein, which were comparable to previously published values for GPX1 activity in Se-adequate rat liver [53,56].

### Growth and body composition

**Figure 1:**
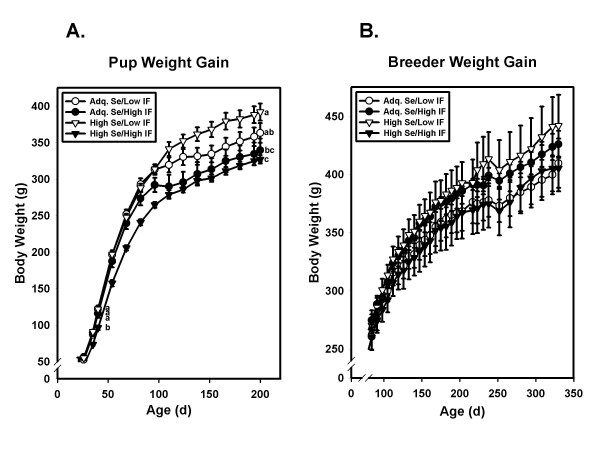
**Weight gain of pups and breeders**. A. Body weights of pups. Symbols and error bars represent means ± SEM for each dietary group at each time point. N = 23–24 rats/group up to 35 days, N = 15–16 rats/group from 35–100 days, and from 100–200 days N = 8 rats/group. By ANOVA, diet was a significant determinant (p < 0.005) of body weight as early as 35 days, which significance continued until the end of the study. At 35 days and 200 days means not sharing a common superscript are significantly (p < 0.05) different by Fisher's pairwise comparisons. The main effect of isoflavones was statistically significant at each time point (p = 0.047 and p = 0.001 at 35 days and 200 days, respectively) while the main effect of Se was not. B. Body weights of breeders. N = 3–4 for each dietary group at each time point. Symbols and error bars represent means ± SEM for each dietary group at each time point. There were no statistically significant effects of diet on body weight at any time point. Abbreviations: Adequate (0.33–0.45 mg/kg diet) Se, Adq. Se; High (3.33–3.45 mg/kg) Se, High Se; Low (10 mg/kg) isoflavones, Low IF; High (600 mg/kg) isoflavones, High IF.

Diet had a significant effect on weight gain of rat pups (Figure 1A). From 35 days until termination of the feeding period the main effect of isoflavones was significant in reducing body weight (p = 0.047 and p = 0.001 at 35 days and 200 days, respectively). Only at the 35 day time point did the main effect of Se approach statistical significance (p = 0.05). In contrast to the effects in pups exposed to their respective treatments from conception, diet had no statistically significant effect on body weight of breeders whose exposure to the diets began after sexual maturity (Figure 1B).

**Figure 2:**
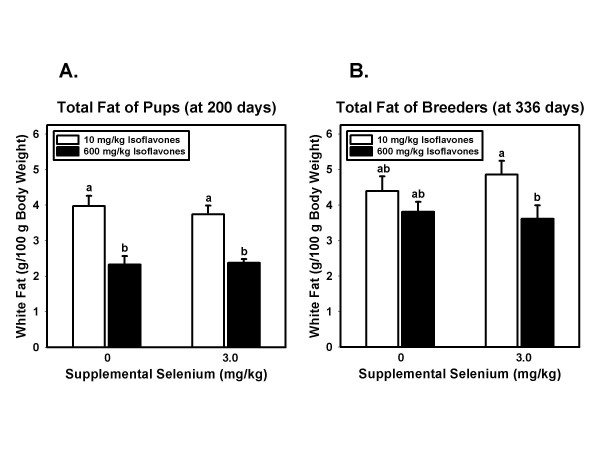
**Body fat of pups and breeders**. A. Body fat, as a percentage of body weight, in pups sacrificed at 200 days of age. Bars and error bars represent means + SEM for each dietary group, N = 8 rats/group. The main effect of isoflavones was highly significant (p < 0.001) while the effect of Se and of the interaction were not. Means not sharing a common superscript are significantly (p < 0.05) different by Fisher's pairwise comparisons. B. Body fat, as a percentage of body weight, in breeders sacrificed at approximately 336 days of age. Bars and error bars represent means + SEM for each dietary group, N = 3–4 rats/group. The main effect of isoflavones was significant (p = 0.038) while the effects of Se and the interaction were not. Means not sharing a common superscript are significantly (p < 0.05) different by Fisher's pairwise comparisons.

Comparable to its effects on body weight, high isoflavone intake was associated with significantly lower body fat in 200 day old rat pups (p < 0.001; Figure 2A). Dietary Se intake had no statistically significant effect on body fat (p = 0.779) nor was the interaction between Se and isoflavones significant. In breeders the main effect of phytoestrogen consumption was significant (p = 0.038) while effects of Se and the interaction were not (Figure 2B).

There were no consistent, significant effects of diet on relative weights (g/100 g body weight) of ventral prostate lobes or of testis in pups at different time points or in breeders.

### Serum hormone concentrations

**Figure 3:**
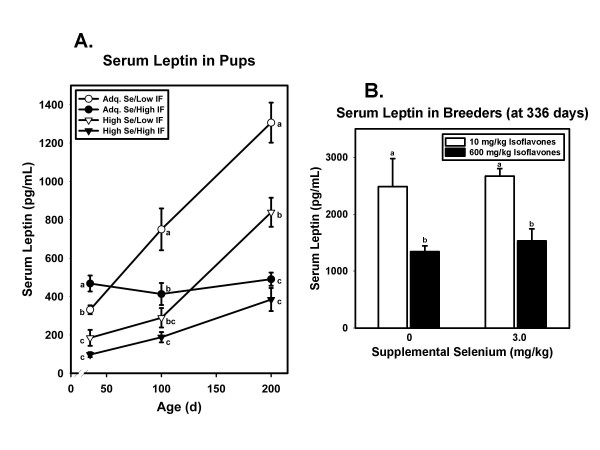
**Serum leptin in pups and breeders**. A. Mean serum leptin concentration in pups at 35, 100, and 200 days of age. Symbols and error bars represent means ± SEM for each dietary group at each time point. At 35 days N = 23–24 rats/group, at 100 days N = 15–16 rats/group, and at 200 days N = 8 rats/group. At each time point, means not sharing a common superscript are significantly (p < 0.05) different by Fisher's pairwise comparisons. At 35 days only Se had a significant effect (p < 0.001) on serum leptin concentrations. At 100 days both Se (p = 0.001) and isoflavones (p = 0.033) had significant main effects. At 200 days only isoflavone intake had a significant main effect (p < 0.001). B. Mean serum leptin concentration in male breeders at 336 days. Only the main effect of isoflavones was statistically significant (p = 0.004). Means not sharing a common superscript are significantly (p < 0.05) different by Fisher's pairwise comparisons.

Serum leptin in pups exposed to diets from conception followed the same pattern as body weight and body fat (Figure 3A). Interestingly, the effects of Se and isoflavones varied over time. At 35 days the main effect of Se on serum leptin levels was significant (p < 0.001) while the effect of isoflavones was not (p = 0.637). At 100 days the main effect of each dietary component was significant (isoflavones, p = 0.033; Se, p = 0.001), with high intake of each being associated with lower leptin levels. By the time rats were 200 days old, the effect of isoflavones was pronounced (p < 0.001) but the effect of Se was no longer significant (p = 0.068). As seen in Figure 3B, the pattern of dietary effects in breeders was similar to that in 200 day old pups. High isoflavone intake significantly (p = 0.004) reduced serum leptin levels, while high Se intake had no effect (p = 0.807).

**Figure 4:**
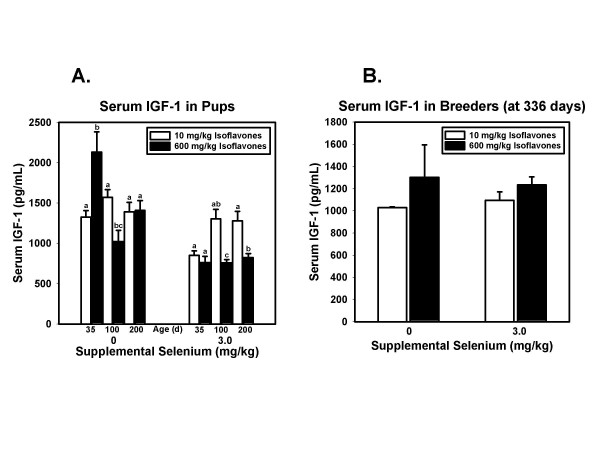
**Serum IGF-1 in pups and breeders**. A. Serum IGF-1 concentrations in pups sacrificed at 35, 100, and 200 days of age. Bars and error bars represent means + SEM for each dietary group, N = 7–8 rats/group. For each time point, means not sharing a common superscript are significantly (p < 0.05) different using Fisher's or Tukey's pairwise comparisons. At each time point the main effects of Se (p < 0.0001, p = 0.022, and p = 0.003 at 35 days, 100 days, and 200 days, respectively) and of isoflavones (p = 0.019, p = 0.022, and p = 0.046 at 35 days, 100 days, and 200 days, respectively) were statistically significant. Interactions were statistically significant at 35 days (p = 0.004) and 200 days (p = 0.034). B. Serum IGF-1 concentrations in male breeders sacrificed at approximately 336 days of age. Neither isoflavones nor Se was significantly associated with serum IGF-1 levels in breeders.

Concentrations of IGF-1 were affected by diet in pups at all time points (Figure 4A) but not in breeders (Figure 4B). At 35 days, effects of Se (p < 0.0001), isoflavones (p = 0.019), and the interaction (p = 0.004) were all statistically significant. At 100 days, the main effects of Se (p = 0.022) and isoflavones (p < 0.0001) were each significant but the interaction was not. At 200 days, Se (p = 0.003) and isoflavones (p = 0.046) each had significant main effects, and the interaction was also significant (p = 0.034). In breeders, neither dietary component significantly affected serum IGF-1 levels.

There were no consistent, significant effects of diet on serum testosterone levels in pups at different time points or in breeders.

## Discussion

The findings in this study of decreased body weight, decreased adiposity, and decreased leptin due to high isoflavone consumption are consistent with most previous reports [46,47,57,58] but at variance with others [59,60]. In the Noble rats used in this study neither high Se nor high isoflavone intake had a consistent, significant effect on serum testosterone levels or prostate weights. In earlier investigations in our laboratories and others, high dietary isoflavone intake either decreased [61,62] or had no effect [63,64] on serum testosterone levels, and reduced [61,64,65] or did not affect [63] prostate weights. These differences in phytoestrogen effects in previous studies may be due, in part, to the different rat strains used in those studies. A recent report [66] showed that Sprague-Dawley rats were less sensitive to phytoestrogens than Fisher 344 rats or CD-1 mice in evaluations of estrogenic activity. To compare results from the present study with those found in previous experiments employing Noble rats, a computer-assisted literature search was conducted. That search found only one published paper presenting results from a study examining soy effects in Noble rats, which showed anti-inflammatory effects in the prostate but no effect on the weights of testis or accessory sex glands [67].

It is of interest to note that pronounced dietary effects seen in rat pups, exposed to the various diets from conception, were in some cases minimal or totally absent in breeders, whose exposure to the diets began after sexual maturity. While this result may be due in part to the relatively small number (3–4) of breeders in each dietary group, similar findings suggesting prenatal imprinting or programming due to diet have been previously reported. Mardon et al. [59] showed that prenatal or perinatal exposure of Wistar rats to phytoestrogens led to a high bone mineral density later in life, compared to animals receiving postnatal exposure. Klein et al. [68] demonstrated long-lasting effects of early exposure to genistein on the immune and endocrine systems of rats. Prepubertal exposure to genistein was shown in other work to increase mammary gland differentiation and suppress DMBA-induced cancer [69].

Besides its effects on serum leptin levels, which decreased with the age of the rat, high dietary Se intake had a statistically significant main effect only on serum IGF-1 levels. The decrease in IGF-1 serum level due to high Se was highly significant in rat pups at 35, 100, and 200 days, but non-significant in breeders. This observation suggests that, like isoflavones, Se may also be involved in prenatal programming or imprinting of later biological response.

Effects of Se on IGF-1 have been examined in comparatively few studies. Chandrasekher and Sailaja [70] found that Se significantly inhibited the ability of IGF-1 to stimulate PI3K activity in cultured lens epithelial cells. More relevant to the present work, Meltzer and Haug [71] fed women daily supplements of 100–300 μg Se as Se-rich bread for 6 weeks, or 400 μg selenomethionine for 15 weeks. Although serum Se levels were significantly increased, no significant changes in IGF-1 were observed. These results were comparable to those seen with breeder rats in this study. Previous work in rats showed that high levels of Se that decreased IGF-1 concentrations also were growth inhibitory [71]. In this work, the main effect of Se on IGF-1 was statistically significant, but no growth effects of Se were observed. This provides clear evidence that, although the supplemented diets provided high levels of Se (3.33–3.45 mg/kg diet), this level was non-toxic to growing Noble rats.

Given the complexity of cancer, with dysfunctional changes at multiple stages and in multiple metabolic pathways, it is perhaps not surprising that the results of clinical trials of single agents have been largely disappointing. This has prompted some investigators to propose a redefinition of "dietary chemoprevention" to focus on combinations and "cocktails" of dietary agents to reduce risk for cancer [72]. Our use in this study of a combination of Se supplements with high isoflavone diets is consistent with this recommendation. It is important to note in this study that in pups exposed to test diets from conception, the combination of high Se with high isoflavones resulted in the lowest weight gain (Figure 1A), the lowest serum leptin (Figure 3A), and the lowest serum IGF-1 concentrations (Figure 4A) of all four dietary treatments. This finding has obvious implications for men in whom prostate cancer risk is increased by higher fat mass [2], higher serum leptin [5-7,73], and/or higher IGF-1 levels [3,4,74,75].

## Conclusion

In summary, high isoflavone intake decreased weight gain, body fat mass, and serum leptin in rat pups exposed to experimental diets from conception. Exposure of breeders to the same diets beginning after sexual maturation produced minimal or no effects. This observation provides support for the possibility of *in utero *imprinting by high isoflavone intake and may call into question the merit of beginning isoflavone supplementation in mature men diagnosed with prostate cancer. High intake of dietary Se was associated in this study with decreased IGF-1 levels in pups prenatally exposed, but not in breeders, suggesting the possibility of prenatal programming by Se, as well. Combining high isoflavones with high Se produced the greatest effects on the parameters measured. This finding provides support for the combination approach to cancer chemoprevention by dietary constituents being advocated by an increasing number of investigators.

## Competing interests

The authors declare that they have no competing interests.

## Authors' contributions

JRT assisted in study design and was responsible for animal feeding and sacrifice, acquisition of data (all measurements but isoflavones), analysis and interpretation of data, and manuscript preparation. EDL assisted in study design with specific reference to diet composition, analysis and interpretation of data, and manuscript preparation. KDRS was responsible for analysis of isoflavone concentrations, and analysis and interpretation of data. DLE was responsible for statistical analysis. MJC obtained funding, oversaw study design, assisted in acquisition of data, analysis and interpretation of data, and manuscript preparation. All authors read and approved the final manuscript.

## Supplementary Material

Additional file 1 **Table 1.** Treatment diets. Click here for file
