# Supplementary material for: Timing of supplementation of selenium and isoflavones determines prostate cancer risk factor reduction in rats
Source: Nutr Metab (Lond). 2008 Nov 10;5:31. doi: 10.1186/1743-7075-5-31 (PMC2615019; doi:10.1186/1743-7075-5-31)
Supplement: Additional file 1 — Table 1. Treatment diets. [file 1743-7075-5-31-S1.doc]

**Table 1: Treatment diets**

**Harlan-Teklad Zeigler Bros.**

**Unit 8604 Phyto. Red. I**

**Nutrient composition**

Protein % 24.48 23.62

Fat % 4.40 5.63

Fiber % 3.69 2.38

Ash % 7.84 6.40

Linoleic acid % 1.87 2.28

**Amino acids**

Arginine % 1.53 1.11

Methionine % 0.42 0.58

Cystine % 0.37 0.27

Histidine % 0.58 0.58

Isoleucine % 1.24 1.17

Leucine % 2.04 2.14

Lysine % 1.46 1·41

Phenylalanine+tyrosine % 1.84 2.05

Threonine % 0.94 0.93

Tryptophan % 0.29 0.25

Valine % 1.26 1.35

**Minerals**

Calcium % 1.36 1.10

Phosphorus % 1.01 0.92

Sodium % 0.29 0.31

Chlorine % 0.49 0.43

Potassium % 1.04 0.55

Magnesium % 0.28 0·17

Iron mg/kg 352.14 354.26

Manganese mg/kg 105.39 99.93

Zinc mg/kg 82.87 61.69

Copper mg/kg 24.42 12.69

Iodine mg/kg 2.46 1.98

Cobalt mg/kg 0.71 0.57

Selenium mg/kg 0.33 0.45

**Vitamins**

Vitamin A IU/g 12.90 6.59

Vitamin D3 IU/g 2.40 7.13

Vitamin E IU/kg 90.18 51.25

Choline mg/g 2.53 1.63

Niacin mg/kg 63.42 69.47

Pantothenic acid mg/kg 21.03 30.67

Pyridoxine (vitamin B6) mg/kg 12.95 9.46

Riboflavin (vitamin B2) mg/kg 7.85 6.66

Thiamine (vitamin B1) mg/kg 27.95 16.35

Menadione (vitamin K3) mg/kg 4.11 3.15

Folic acid mg/kg 2.72 3.15

Biotin mg/kg 0.39 0.29

Vitamin B12 mg/kg 51.20 47.92

Vitamin C mg/kg 0.00 0.00

The ingredients list (first four) for the Harlan-Teklad 8604 diet: soybean

meal, wheat middlings, flaked corn, ground corn; for the Zeigler Bros. Phyto. Red. I diet: corn, wheat, wheat middlings, NIH fish meal.
